# Ultrasound Shear Wave Elastography Evaluation of the Liver and Implications for Perioperative Medicine

**DOI:** 10.3390/jcm13133633

**Published:** 2024-06-21

**Authors:** Giancarlo Suffredini, Wei Dong Gao, Jeffrey M. Dodd-o

**Affiliations:** Department of Anesthesiology and Critical Care Medicine, Division of Cardiac Anesthesia, Johns Hopkins University School of Medicine, Baltimore, MD 21205, USA; wgao3@jhmi.edu (W.D.G.); jdoddo@jhmi.edu (J.M.D.)

**Keywords:** liver stiffness, shear wave elastography, perioperative medicine, outcomes

## Abstract

Ultrasound shear wave elastography (SWE) is a non-invasive, low risk technology allowing the assessment of tissue stiffness. Used clinically for nearly two decades to diagnose and stage liver fibrosis and cirrhosis, it has recently been appreciated for its ability to differentiate between more subtle forms of liver dysfunction. In this review, we will discuss the principle of ultrasound shear wave elastography, its traditional utilization in grading liver cirrhosis, as well as its evolving role in identifying more subtle degrees of liver injury. Finally, we will show how this capacity to distinguish nuanced changes may provide an opportunity for its use in perioperative risk stratification.

## 1. Introduction

Changes in stiffness have long been recognized to accompany common pathologic processes, including inflammation, malignancy, fibrosis, and congestion. These changes were historically assessed by clinicians through manual palpation [1]. Shear wave elastography is an ultrasound-based imaging technique that permits the non-invasive measurement of tissue stiffness in a quantifiable and reproducible manner. The objectives of this review are to discuss the basic principles of ultrasound shear wave elastography, to review its utility in evaluating hepatic and cardiac health, to examine its role in perioperative risk stratification, and to consider how it may inform interventions to decrease perioperative risk.

## 2. Principles of Ultrasound Shear Wave Elastography

A force is typically described in terms of the energy vector perpendicular to the surface of a material. By contrast, a shear force is an energy vector applied parallel to the surface of a material. The ability of a material to withstand changes in length when subjected to shear force is an inherent property of each material. This property is mathematically described by Young’s modulus, which is the ratio of “the applied force per unit area (a.k.a. stress)” divided by “the resultant change in tissue dimension (a.k.a strain)”. It is expressed in units of pressure called kilopascals (kPa). Shear wave elastography aims to quantitatively image the Young’s modulus in biologic tissue. In shear wave elastography, a force is applied perpendicular to tissue and results in the generation of shear waves. The imaging system then tracks the velocity of the induced shear waves and uses this to calculate the Young’s modulus [2,3,4].

**Figure 1:**
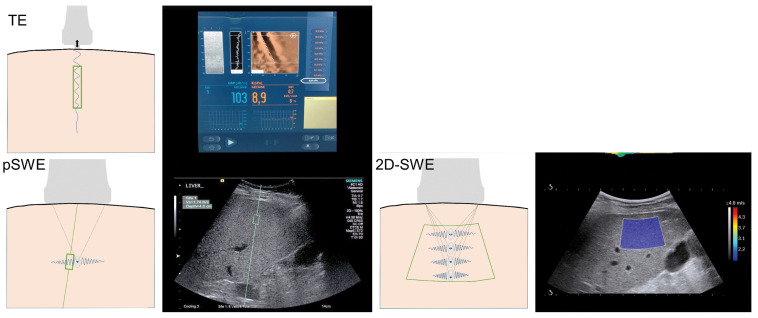
Illustrations of US elastography techniques, including transient elastography (FibroScan, Echosens, Paris, France), point shearwave elastography (Virtual Touch Quantification, Siemens Acuson S2000, Munich, Germany), and 2D-SWE (Aixplorer, Supersonic Imagine, Aix-en-Provence, France). Sampling area for each method is depicted by enclosed green area. TE and pSWE have a fixed sampling area size, though pSWE allows the depth and location to be chosen. Two-dimensional SWE has the ability of pSWE sampling area placement with the additional ability to change the size. TE = transient elastography, pSWE = point shearwave elastography, 2D-SWE—two dimensional shear wave elastography. Reproduced with permission from “Quantitative Elastography Methods in Liver Disease: Current Evidence and Future Directions”, by Kennedy, P. et al. 2018 [7].

To generate shear waves in an object, current commercial shear wave measuring systems first transmit force to the object by using either a mechanical piston or ultrasound waves. The force is applied perpendicular to the surface of the skin, is transmitted to the object of interest, and a portion of this force then dissipates parallel to the surface of the object as a shear wave. The velocity of this shear wave is then measured and used to calculate Young’s modulus. In vibration controlled transient elastography (VCTE), the applied perpendicular force is generated with a mechanical piston that is attached to a single-element ultrasound transducer. In contrast, point shear wave (pSWE) and 2D-shear wave elastography (2D-SWE) use sound energy to generate the applied perpendicular force. In each case, the transducer then tracks the velocity of the shear waves using pulse-echo sequences and reports the values [5]. Shear wave speed measurement is either taken from one small fixed area (1 × 0.5 cm^2^) as in pSWE, a larger (2 × 3 cm^2^) modifiable area as in 2D-SWE, or a cylindrical 3 cm^3^ area in VCTE [6]. Unlike VCTE, both pSWE and 2D-SWE offer the advantage of providing a conventional B-mode image of the tissue being interrogated. This image allows the user to select a region of interest for interrogation (Figure 1).

## 3. Clinical Applications of Shear Wave Elastography

### 3.1. Liver Fibrosis and Inflammation

Fibrosis is a common pathway to organ injury and failure, and its effects on the heart, lungs, kidney, and liver are well described [8,9,10,11]. Here, a persistent inflammatory response leads to structural changes in the extracellular matrix, increased extracellular matrix deposition, parenchymal cell death, disrupted architecture, and eventually loss of organ function [8,12]. Early structural changes of the extracellular matrix include enhanced cross-linking of collagen, which results in an elevated matrix stiffness. Matrix stiffness is now recognized as a critical regulator of tissue fibrogenesis that alters the normal wound-healing response and promotes organ fibrosis [13,14].

**Figure 2:**
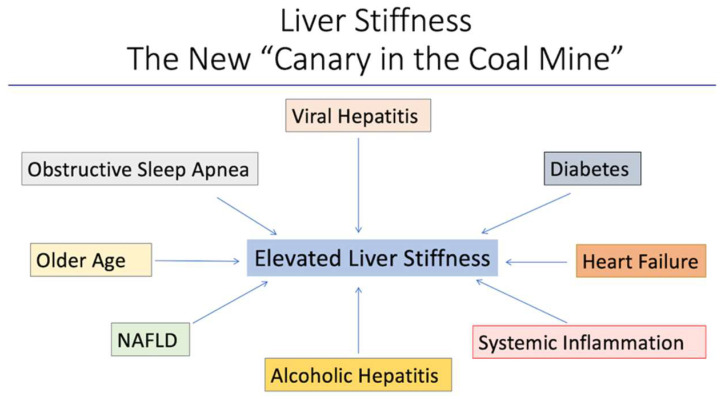
Elevations in liver stiffness may serve as an early warning of organ damage related to multiple causes. Early recognition may help to identify those patients in need of more aggressive therapy.

Regardless of the underlying cause, repetitive injury to the liver leads to fibrosis and eventually cirrhosis. If the stimulus for fibrosis is eliminated early enough, reversal of fibrosis can occur. In cirrhosis, regression (improvement, but not reversal) is possible and improves clinical outcomes [12]. Historically, the primary method of assessing and/or confirming the degree of liver fibrosis was with liver biopsy. By providing a non-invasive method of evaluating liver fibrosis, shear wave elastography may facilitate early identification at a stage when reversibility is still possible. Furthermore, elevated liver stiffness in the absence of organic liver disease may serve as an early warning of organ damage related to a variety of causes [15,16,17,18,19] and facilitate appropriate interventions to minimize disease progression (Figure 2).

The first commercially available shear wave elastography machines used VCTE to assess tissue stiffness. They were primarily used for assessing liver stiffness, and there is therefore an abundance of literature relating liver stiffness to the histologic staging of liver fibrosis.

An early example is a 2006 study evaluating the relationship between liver stiffness and advanced liver disease of various etiologies in over 700 patients [20]. Half of the enrolled patients had concomitant liver biopsies. In this study, shear stiffness values of 7.2 kPa, 12.5 kPa, and 17.6 kPa predicted moderate fibrosis, severe fibrosis, and cirrhosis, respectively, with a positive predictive value of at least 90% in each case. The corresponding areas under the receiver operator characteristic for moderate fibrosis, severe fibrosis, and cirrhosis were 0.8, 0.9, and 0.96, respectively. Liver stiffness was good at detecting moderate degrees of fibrosis and was excellent at detecting severe fibrosis and cirrhosis.

A decade later, liver stiffness values were included in consensus guidelines to help identify and risk stratify patients with silent advanced liver disease [21]. Here, liver stiffness values less than 10 kPa in the absence of other risk factors ruled out advanced liver disease, and values greater than 15 kPa were highly suggestive of advanced liver disease. These numbers were later refined in a cohort of over 5500 patients with chronic liver disease, where all the patients had a liver biopsy with a liver stiffness scan, providing validation of the degree of liver stiffness with histologic staging of liver disease [22]. Liver stiffness less than 8 kPa effectively ruled out advanced fibrosis, whereas liver stiffness greater than 12 kPa ruled in advanced liver fibrosis.

The prevalence of silent advanced fibrosis in the general population was unknown prior to non-invasive liver evaluation with elastography. Elastography identifies advanced liver fibrosis in 5–7% of the general population [23]. The risk factors most associated with elevated liver stiffness are diabetes, obesity, hypertriglyceridemia, metabolic syndrome, and excessive alcohol consumption [23]. Metabolic risk factors (i.e., diabetes, obesity, hypertriglyceridemia, and metabolic syndrome) have a dramatic effect on the prevalence of elevated liver stiffness in the general population [24]. The prevalence of an elevated stiffness rises from 0.4% in those persons without metabolic risk factors to 5% in those with one or more metabolic risk factors.

**Figure 3:**
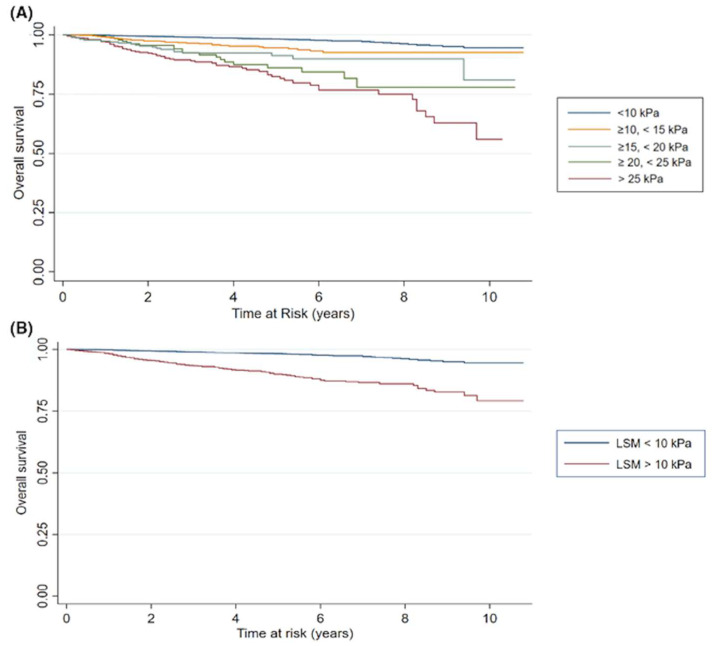
Liver stiffness is a predictor of all-cause mortality in people with non-alcoholic fatty liver disease. (**A**) Kaplan–Meier of liver stiffness measurement (LSM) (kPa) relative to survival using the rule of 5 s threshold values (blue bar = LSM < 10 kPa, orange bar = LSM ≥ 10 and <15 kPa, teal bar = LSM ≥ 15 kPa and <20 kPa, green bar = LSM ≥ 20 and <25 kPa, red bar = LSM ≥ 25 kPa). (**B**) Kaplan–Meier of LSM (kPa) based on no cACLD (blue bar, <10 kPa), or suggestive cACLD (red bar = LSM ≥ 10 kPa). Reproduced with permission from “Liver stiffness (Fibroscan^®^) is a predictor of all cause mortality in people with non-alcoholic fatty liver disease”, by Braude, M. et al. 2022 [25].

The impact of silent liver disease can be seen in a recent study involving over 7000 patients with non-alcoholic fatty liver disease (NAFLD), where a liver stiffness value greater than 10 kPa was shown to confer a substantial mortality risk over a 10 year period (Figure 3). The mortality risk increased with higher liver stiffness, and the most common causes of death in the cohort were non-liver malignancy, sepsis, liver disease, and cardiac disease [25].

### 3.2. MAFLD/MASLD

The last few years have seen a transition in the nomenclature regarding steatotic liver disease. Metabolic dysfunction-associated fatty liver disease (MAFLD) and, more recently, metabolic dysfunction-associated steatotic liver disease (MASLD) have been proposed to replace NAFLD. A diagnosis of NAFLD required the presence of hepatic steatosis without concurrent liver disease. Although many of these patients had concomitant obesity, type 2 diabetes, and metabolic risk factors including hypertension, poor cholesterol profiles, and elevated triglycerides, these were not required for the diagnosis of NAFLD. In addition, the presence of concomitant risk factors for liver disease, such as viral hepatitis and alcohol use, excluded the diagnosis of NAFLD.

The presence of hepatic steatosis remains a core defining feature of MAFLD. A diagnosis of MAFLD, however, requires the presence of either type 2 diabetes, obesity, or being lean/normal weight with at least two metabolic risk factors [26]. Importantly, this classification scheme does not eliminate the presence of concurrent liver disease in the diagnosis, including viral hepatitis or alcohol use [27]. When compared to NAFLD, MAFLD has been shown to improve the detection of elevated liver stiffness [28] and to better predict the progression of atherosclerotic cardiovascular risk [29]. Additionally, the risk of cardiovascular events has been shown to be higher in patients diagnosed with MAFLD when compared to those diagnosed with NAFLD [30].

MASLD is a more recent classification of what was previously called NAFLD [31]. A diagnosis of MASLD requires the presence of hepatic steatosis along with one cardiometabolic risk factor. A subcategory of MASLD accounts for the presence of increased alcohol intake (MetALD). However, steatotic liver diseases caused by other etiologies (i.e., alcohol-related liver disease, drug induced liver injury, monogenic disease, viral hepatitis, and cryptogenic) remain discretely categorized and are not defined as MASLD [32]. The elimination of concurrent liver disease in a diagnosis of MASLD is the major differentiation from a diagnosis of MAFLD. Despite the requirement of having at least one metabolic risk factor, the clinical profiles, mortality rates, and natural history of those with MASLD are nearly identical to those with NAFLD [33,34].

Regardless of the classification scheme, an elevated liver stiffness in patients with either known steatotic liver disease or the presence of metabolic risk factors should trigger consideration for the evaluation of underlying cardiovascular disease [35]. As the population of patients with metabolic-related liver disease grows, ultrasound elastography evaluation of the liver will become more common, and it is essential that clinicians caring for these patients in the perioperative arena understand the significance of the relationship between an elevated liver stiffness and underlying cardiovascular disease.

## 4. Liver Stiffness and Symptomatic Post Hepatectomy Liver Failure

Hepatocellular carcinoma is a primary malignancy of the liver that is highly associated with chronic liver disease and cirrhosis. For patients who do not have cirrhosis and have smaller tumors, surgical resection is the treatment of choice, where major resections can generally be performed safely [36]. Liver function has typically been assessed using the Child–Pugh classification. The Child–Pugh classification of severity of liver disease is according to the degree of ascites, the serum concentration of bilirubin and albumin, the prothrombin time or international normalized ratio, and the degree of encephalopathy. A score of 5–6 (class A) is considered well compensated disease, 7–9 (class B) is considered significant functional compromise, and 10–15 is considered decompensated disease. Child–Pugh class A may encompass a spectrum of patients, from those with normal liver function to those with compensated cirrhosis, and therefore limits its predictive power for post-operative outcomes. However, liver stiffness in patients classified as Child–Pugh A severity, has been shown to stratify the risk of post-operative hepatic failure in patients with both large and small liver resections [37,38,39]. The ability of liver stiffness to risk stratify among patients considered to be low risk may be due to elevated stiffness indicating significant portal hypertension or indicating the effects of concomitant metabolic diseases.

## 5. The Hepatic Cardiac Axis

**Figure 4:**
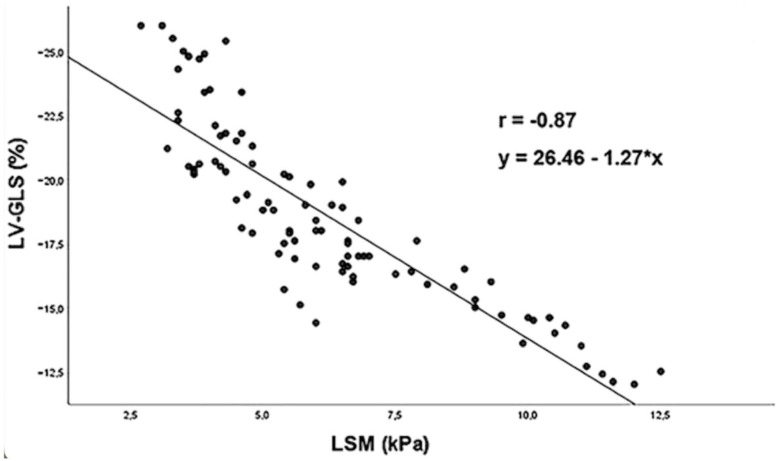
Corrrelation between LSM and LV-GLS (r = −0.87). Reproduced with permission from Sonaglioni, A. et al. 2022 [43].

The interaction between heart disease and liver disease is complex. Both share many risk factors, and several studies have shown that heart disease often accompanies elevated liver stiffness [35,40,41,42]. A recent study evaluated the presence of subclinical heart disease in patients with non-advanced non-alcoholic fatty liver disease (NAFLD) [43]. In just under 100 patients with non-advanced NAFLD, heart function was measured using a comprehensive echocardiographic examination assessing systolic and diastolic function as well as biatrial and biventricular myocardial strain. Despite normal biventricular systolic function and mild diastolic dysfunction, left ventricular global longitudinal strain (LV-GLS) was impaired in two-thirds of the patients. In a multivariate logistic regression analysis, liver stiffness (OR 9.26) was a strong independent predictor of reduced LV-GLS as well as having a very strong inverse correlation with LV-GLS (r = −0.87) (Figure 4). NAFLD patients with liver stiffness greater than 5.5 kPa have a significantly increased probability of concomitant subclinical myocardial dysfunction, as reflected by a reduced LV-GLS [43].

## 6. Congestive Heart Failure

Disease processes that lead to an increase in the central venous pressure may increase the pressure of the inferior vena cava (IVC) and the hepatic veins. The liver is enclosed within a rigid capsule and has a venous system that has no valves to protect it from the increased pressures of the IVC. Thus, elevated IVC pressures rapidly result in hepatic congestion and increased liver stiffness. In patients with heart disease demonstrated clinically as heart failure, liver stiffness has therefore been used to risk stratify patients who are without known primary liver disease.

The influence of central venous pressure on liver stiffness was first demonstrated in an animal model where liver stiffness measurements were taken before, during, and after clamping of the IVC [44]. The authors found that liver stiffness during clamping of the IVC, as assessed by VCTE, dramatically increased. After the release of the clamp, the stiffness returned to near-baseline values. This demonstrated that CVP could control liver stiffness in a reversible manner [44]. In clinical applications, liver stiffness as assessed by elastography has been shown to reliably estimate CVP [45,46].

**Figure 5:**
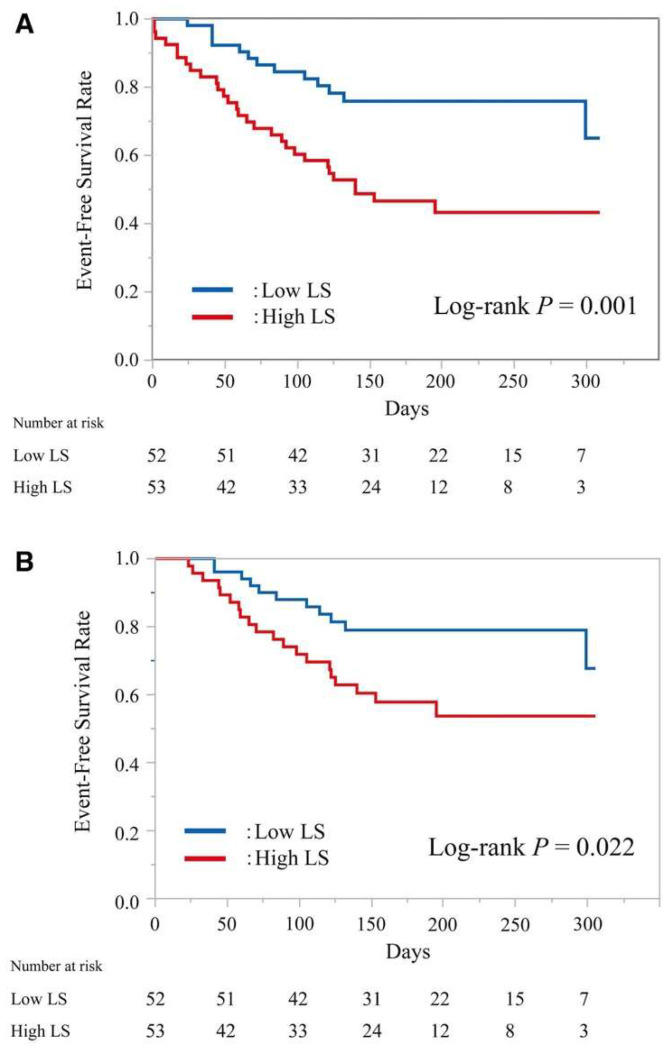
Kaplan–Meier plot of event-free (cardiovascular death or readmission for heart failure) survival (**A**) and Kaplan–Meier plot of event-free (readmission for heart failure) survival (**B**), in patients in the high liver stiffness (LS) (≥8.8 kPa, red line) and low LS (<8.8 kPa, blue line) groups. Reproduced with permission from Saito et al. 2018 [49].

The ability of liver stiffness to predict adverse outcomes in patients with heart failure has been demonstrated in several studies [17,46,47,48]. In one example, 105 patients with acute decompensated heart failure were divided at hospital admission into two groups based on median liver stiffness: Those with a liver stiffness measurement greater than 8.8 kPa and those with a liver stiffness measurement (LSM) less than 8.8 kPa. Within a median follow-up period of 153 days from admission, death or readmission for heart failure had occurred in 54% of patients with LSM > 8.8 kPa and only 25% of patients with LSM < 8.8 kPa (*p* = 0.001). After adjusting for age, sex, and indices related to organ congestion, the patients with a high liver stiffness on admission were significantly more likely to experience death from cardiovascular disease or readmission for heart failure (HR: 2.57) over the follow-up period of just under one year [49] (Figure 5).

In another study of 171 patients, patients admitted to the hospital with acute decompensated heart failure were treated with goal-directed medical therapy and then had a liver stiffness measurement performed at the time of discharge [17]. Patients were stratified into tertiles based on liver stiffness values: Group 1 < 4.7 kPa, Group 2 < 4.7–6.9 kPa, and Group 3 > 6.9 kPa. Patients in Group 3 were in the advanced New York Heart Association functional class and had a significantly higher risk of death or hospital readmission for heart failure when compared to the other two groups (log-rank test: *p* < 0.001, for first vs third tertiles; follow-up median 203 days; follow-up IQR 67–429 days). In addition, univariate Cox regression analysis showed a significant predictive value for cardiac events with a hazard ratio of 1.13 per 1 kPa increase in LSM. This suggests that, in patients treated for acute decompensated heart failure, liver stiffness measurement at the time of discharge is a useful index for identifying those with persistent advanced heart failure and those at risk for readmission or death within the next 18 months.

## 7. Overview of Perioperative Risk for Non-Emergent Cardiac Surgery—Society of Thoracic Surgeons (STS) Risk Calculator

The Society of Thoracic Surgeons offers an operative risk calculator to inform patients and their surgeons of the risks facing the patient [50]. The calculated risks include operative mortality, morbidity and mortality, stroke, renal failure, reoperation, prolonged ventilation, deep sternal wound infection, and postoperative length of hospital as long (>14 days) or short (<6 days). These risks are specific to eight types of planned surgery: Isolated coronary artery bypass grafting, isolated aortic valve replacement, isolated mitral valve replacement, isolated mitral valve repair for any etiology, isolated mitral valve repair for primary mitral regurgitation, concomitant coronary artery bypass surgery and aortic valve replacement, concomitant coronary bypass surgery and mitral valve replacement, and concomitant coronary artery bypass grafting with mitral valve repair [50]. The risk calculator addresses many patient risk factors and comorbidities with high granularity; however, liver disease is addressed only in very general terms. Thus, liver disease is only considered positive if a patient has a known history of hepatitis B, hepatitis C, autoimmune hepatitis, drug induced hepatitis, cirrhosis, esophageal varices, liver transplant, or congestive hepatopathy. Furthermore, non-alcoholic steatohepatitis (NASH) is excluded in the absence of cirrhosis. This focus on advanced liver disease as the only liver morbidity that influences outcome following cardiac surgery likely reflects the literature suggesting that mortality following cardiac surgery is associated with hepatic dysfunction only if it is significant hepatic dysfunction. In contrast to perioperative mortality, however, perioperative morbidity is associated with more subtle changes in hepatic function [51,52,53]. More specifically, total hospital lengths of stay and renal failure requiring dialysis can each vary by 60–80% among different degrees of Child–Pugh Class A and B liver disease [52].

## 8. Perioperative Risk Stratification by Assessment of Liver Stiffness—How Does It Inform Interventions That Decrease Perioperative Risk

A prospective study of over 100 patients suggests that preoperative liver stiffness measured with shear wave elastography may help identify patients at risk for non-lethal morbidity following cardiac surgery [54]. After adjusting for baseline characteristics, surgery type, and time on cardiopulmonary bypass, an elevated preoperative liver stiffness (>9.5 kPa) was associated with a significantly longer hospital length of stay when compared to those patients with a normal or moderately elevated liver stiffness measurement. In addition, although 40% of patients in the low or moderately elevated liver stiffness groups experienced short hospital lengths of stay (<6 days), no patients in the high stiffness group left the hospital in less than 6 days. Importantly, only 2 patients had any prior record of liver disease. Both patients endorsed a known history of fatty liver disease, with one of them also having an incidental finding of portal hypertensive gastropathy on a computed tomography scan.

In addition to identifying patients at risk for non-lethal morbidity following cardiac surgery, liver elastography may also prove to be a useful tool in identifying early liver disease at a point at which it is relatively easily reversible. Liver stiffness from non-alcoholic fatty liver disease (NAFLD) can be substantially lowered by modest weight loss and exercise [55]. For example, consumption of more than 3 cups of coffee per day has been shown to be protective against a liver stiffness of 9.5 kPa or greater [56]. By contrast, consumption of more than 2 sugar-sweetened beverages per day has been associated with a liver stiffness greater than 7 kPa [57]. Though a reduction in liver stiffness can be accomplished by lifestyle modifications, it requires substantial lead time and would be ineffective if implemented in the immediate perioperative period.

Elevated liver stiffness as measured by elastography may also reflect the salutary effects of specific pharmacotherapies. Incretins (ex GLP-1 receptor agonists; GLP-1a) are gut-derived peptide hormones that are rapidly secreted in response to a meal. They reduce appetite and body weight, improve insulin sensitivity, have antioxidant activity, promote an anti-inflammatory milieu, and improve mitochondrial health [58]. In a retrospective study of patients with type 2 diabetes, those taking GLP-1a had a lower risk of liver stiffness greater than 8 kPa compared to a historic cohort who were not taking GLP-1a medications (adjusted odds ratio 2.14) [59].

In a placebo-controlled trial, the GLP-1a semaglutide was shown to reduce liver stiffness by up to 30% after 52 weeks of treatment. This reduction in liver stiffness was associated with the resolution of liver inflammation but not the grade of liver fibrosis. A similar capacity to improve liver inflammation, but not necessarily liver fibrosis, is seen when hepatitis induced by viral infection (hepatitis C) is resolved pharmacologically [60].

More aggressive incretin therapy may offer a more effective reduction in liver stiffness, along with its associated morbidities. Retatrutide is a triple hormone receptor agonist, targeting three of the incretin receptors: GLP-1, GIP, and glucagon receptors. In a phase 2 trial, retatrutide was found to reduce total body weight by 25% at 48 weeks, with up to 5% of body weight reduction seen at only 4 weeks [61]. Although this study did not evaluate changes in liver stiffness, liver stiffness tends to parallel weight loss in patients with obesity or insulin resistance. If this holds true, the short lag time between retatrutide initiation and hepatic stiffness could indicate a feasible pharmacologic option for perioperative implementation.

## 9. Conclusions

Shear-wave elastography is a sensitive indicator of liver injury from various causes. Though its capacity to identify etiology is limited, it appears to offer granularity in distinguishing the degree of injury. It therefore may offer a non-invasive tool to identify injury at a time when it is amenable to lifestyle, dietary, and/or pharmacologic interventions. Such interventions may even be useful in reducing perioperative morbidity.
